# Bilateral Intercostal Cryoanalgesia After Sternotomy for Coronary Artery Bypass Grafting

**DOI:** 10.3390/medsci14050510

**Published:** 2026-08-24

**Authors:** Shahzad G. Raja, Amina Khalil, Jezerene Ronquillo, Maria Alberici, Charlotte Sear, Katarina Lenartova, Nandor Marczin

**Affiliations:** 1 Department of Cardiac Surgery, Harefield Hospital, Hill End Road, London UB9 6JH, UK; amina.khalil2@nhs.net; 2 Department of Pain Management, Harefield Hospital, Hill End Road, London UB9 6JH, UK; j.ronquillo@nhs.net (J.R.); charlotte.sear1@nhs.net (C.S.); katarina.lenartova@nhs.net (K.L.); 3 Department of Anaesthesia and Critical Care, Harefield Hospital, Hill End Road, London UB9 6JH, UK; maria.alberici@nhs.net

**Keywords:** bilateral intercostal cryoanalgesia, coronary artery bypass grafting, enhanced recovery after cardiac surgery, median sternotomy, opioid-sparing analgesia, postoperative pain, health economics

## Abstract

Background: Effective opioid-sparing analgesia after median sternotomy remains an unmet need in cardiac surgery. We evaluated whether bilateral intercostal cryoanalgesia, added to standard multimodal analgesia, was associated with postoperative pain, opioid consumption, recovery and early hospital-resource use after isolated coronary artery bypass grafting (CABG). Methods: This single-centre, non-randomised comparative service evaluation included 60 patients undergoing isolated CABG through median sternotomy between 1 November 2025 and 30 May 2026 (30 cryoanalgesia; 30 standard care). The prespecified primary endpoint was cumulative movement-evoked pain burden, quantified as the area under the curve (AUC) for scores recorded on postoperative days 1–3. Secondary endpoints included rest-pain AUC, opioid consumption expressed as oral morphine equivalents (OME), time to first bowel opening, postoperative length of stay and an exploratory break-even calculation. Results: Cryoanalgesia was associated with lower movement-pain AUC (adjusted mean difference −3.56 score-days, 95% confidence interval [CI] −4.62 to −2.50; *p* < 0.001) and rest-pain AUC (−3.16 score-days, 95% CI −4.19 to −2.14; *p* < 0.001). Total observed opioid consumption was lower by 112.9 mg OME (95% CI −192.8 to −33.1; *p* = 0.006), length of stay by 1.32 days (95% CI −1.93 to −0.71; *p* < 0.001), and time to first bowel opening by 0.49 days (95% CI −0.92 to −0.06; *p* = 0.027). Conclusions: In this small non-randomised evaluation, bilateral intercostal cryoanalgesia was associated with lower early pain and opioid exposure and faster recovery. Selection, temporal and residual confounding preclude causal or cost-effectiveness conclusions. Prospective randomised evaluation with fixed observation periods and longer-term safety follow-up is required.

## 1. Introduction

Median sternotomy remains the standard surgical approach for coronary artery bypass grafting (CABG) despite continued evolution in operative techniques, conduit strategies and perioperative care [1,2]. Although contemporary cardiac surgery has achieved excellent operative outcomes, postoperative pain following sternotomy continues to represent a significant clinical challenge [3,4]. Inadequately controlled pain impairs effective coughing, deep breathing and chest physiotherapy, delays mobilisation, contributes to pulmonary complications and prolongs hospitalisation [3,4]. Furthermore, poorly controlled acute postoperative pain is an established risk factor for persistent post-sternotomy pain, which affects a substantial proportion of patients and may adversely influence long-term quality of life [5].

Despite these recognised consequences, postoperative analgesia following cardiac surgery continues to rely predominantly on systemic opioids. Intravenous patient-controlled analgesia remains widely used after CABG, frequently supplemented with paracetamol and non-opioid analgesics as part of multimodal analgesic regimens [3,6]. Although effective, opioid-based analgesia is associated with well-recognised adverse effects including postoperative nausea and vomiting, ileus, constipation, urinary retention, sedation, respiratory depression and delayed mobilisation [7]. More recently, increasing awareness of persistent postoperative opioid exposure has focused attention on strategies that minimise perioperative opioid requirements while maintaining effective analgesia [8,9].

In contrast, thoracic surgery has undergone a profound transformation in perioperative pain management. The introduction of enhanced recovery pathways, early extubation and regional analgesic techniques has shifted postoperative care away from opioid-centred strategies towards multimodal, opioid-sparing analgesia [10,11]. Epidural analgesia, paravertebral blockade, erector spinae plane blocks and, more recently, cryoanalgesia have all demonstrated favourable effects on postoperative recovery following thoracic procedures [12,13,14,15]. Consequently, cryoanalgesia has become an established adjunct for pain management in selected thoracic surgical populations because it provides prolonged analgesia without the need for indwelling catheters or continuous local anaesthetic infusions [15].

Whether these advances can be translated to cardiac surgery remains uncertain. Historically, median sternotomy has often been regarded as less painful than thoracotomy. However, this perception may reflect differences in perioperative management rather than differences in nociceptive injury [16]. Thoracic surgical patients are frequently extubated immediately after surgery and routinely receive regional analgesia, whereas cardiac surgical patients have traditionally remained sedated, mechanically ventilated and treated with systemic opioids during the period of greatest nociceptive input [10,17]. As fast-track cardiac anaesthesia and enhanced recovery programmes become increasingly adopted, postoperative pain following sternotomy is becoming more clinically relevant because patients are awakened and mobilised substantially earlier. Consequently, effective regional analgesic strategies that reduce opioid exposure without compromising safety are likely to assume increasing importance [9].

Cryoanalgesia produces a reversible axonotmesis through rapid cooling of peripheral nerves, resulting in temporary interruption of nociceptive transmission while preserving the endoneurial architecture and permitting predictable axonal regeneration over subsequent weeks [18]. Unlike fascial plane blocks, cryoanalgesia is performed under direct vision, requires no ultrasound guidance, avoids indwelling catheters and provides sustained analgesia without continuous local anaesthetic administration [19]. These characteristics make it particularly attractive during cardiac surgery, where concerns regarding anticoagulation and catheter-related complications may limit wider adoption of some regional anaesthetic techniques [20].

To our knowledge, bilateral intercostal cryoanalgesia performed during CABG through median sternotomy has not been systematically evaluated. We hypothesised that bilateral cryoanalgesia of the second through seventh intercostal nerves would reduce cumulative postoperative pain, decrease opioid requirements, facilitate recovery and shorten hospital stay compared with standard multimodal analgesia alone. Accordingly, we undertook a comparative service evaluation to assess the clinical effectiveness and early economic implications of incorporating bilateral intercostal cryoanalgesia into routine perioperative care for patients undergoing isolated CABG through median sternotomy.

## 2. Methods

### 2.1. Study Design

This single-centre, non-randomised comparative service evaluation was conducted at a tertiary cardiac surgery centre between 1 November 2025 and 30 May 2026. All patients included in the analytic cohort underwent surgery during this overall study period. Cryoanalgesia was introduced prospectively as a service-improvement adjunct; treatment was not randomly assigned and the clinical record used for this evaluation did not document a uniform allocation rule. The intervention cohort comprised 30 eligible patients who received and consented to cryoanalgesia during the implementation period, and the comparator comprised 30 eligible patients managed with the pre-existing multimodal pathway during the same overall study period. Group-specific enrolment dates were not available; therefore, the cohorts cannot be characterised as definitively sequential or concurrent. The target of 30 patients per group was a pragmatic fixed service-evaluation sample rather than the result of a formal power calculation. Because patient acceptance, availability of the device and trained operators, and clinical scheduling could influence receipt of cryoanalgesia, selection, implementation and temporal bias cannot be excluded.

Cryoanalgesia was introduced as a service-improvement initiative using a device already employed in thoracic surgical practice. The project was formally registered within the institution’s clinical-governance framework as a service evaluation (Synbiotix Project ID: 19721); under that determination, formal Research Ethics Committee review was not required. Written procedure-specific consent was obtained from intervention patients. Comparator information comprised anonymised routinely collected clinical data analysed under the approved service-evaluation framework, for which additional research consent was not required. The evaluation was conducted in accordance with the principles of the Declaration of Helsinki and is reported in accordance with the Strengthening the Reporting of Observational Studies in Epidemiology statement [21].

### 2.2. Patient Population

All adult patients undergoing isolated CABG through a standard median sternotomy during the study period were screened. Receipt of cryoanalgesia was determined during clinical implementation rather than by random allocation. Patients treated with bilateral intercostal cryoanalgesia in addition to standard multimodal analgesia constituted the intervention cohort; a separately selected sample treated with standard multimodal analgesia alone formed the comparator cohort. The available evaluation dataset recorded the common overall study period but did not retain group-specific enrolment dates or complete surgeon-level allocation data; therefore, the cohorts cannot be characterised as definitively concurrent or sequential, and temporal and provider effects could not be modelled.

Patients undergoing concomitant cardiac procedures, redo sternotomy, minimally invasive CABG or emergency surgery, and those with pre-existing chronic pain were excluded. Fifteen otherwise eligible patients who declined the intervention-specific consent were not included in either analytic cohort; they were not reassigned to the comparator cohort because the comparator had been defined independently as a separately selected standard-care sample. This exclusion may have introduced consent-related selection bias.

**Figure 1:**
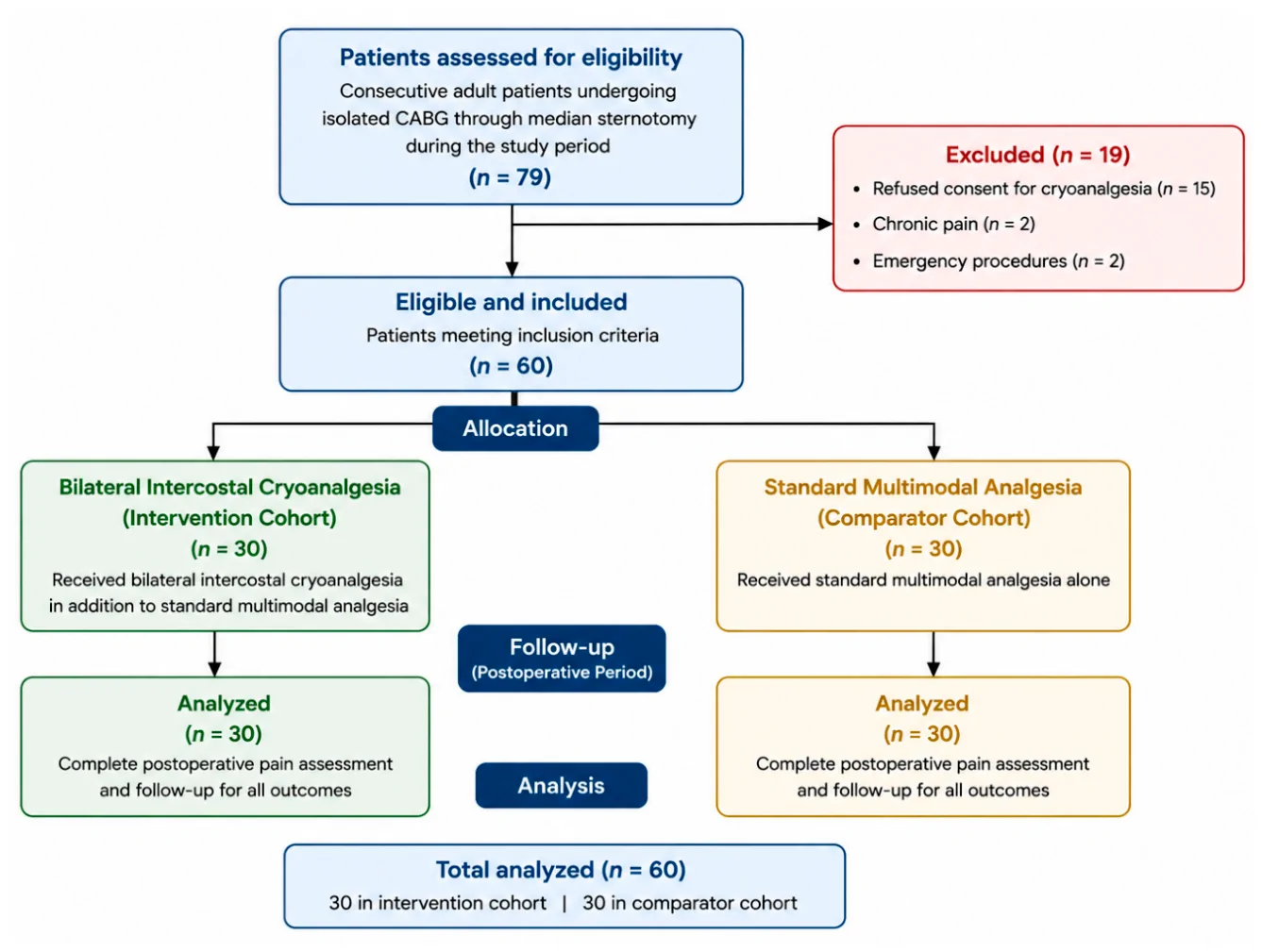
Patient eligibility and allocation flowchart.

A total of 79 patients undergoing isolated CABG through median sternotomy were screened. Nineteen were excluded: 15 declined intervention-specific consent, two had pre-existing chronic pain and two underwent emergency surgery. The analytic sample comprised 30 patients who received cryoanalgesia and a separately selected sample of 30 patients who received standard care (Figure 1). Group-specific enrolment dates were unavailable.

Baseline demographic characteristics, cardiovascular risk factors, comorbidities and perioperative variables were prospectively collected from the institutional cardiac surgical database and electronic medical records. Baseline patient characteristics are summarised in Table 1.

### 2.3. Operative Technique

All procedures were performed through a standard median sternotomy using contemporary techniques of myocardial protection and coronary revascularisation. Bilateral internal mammary arteries with endoscopically harvested long saphenous vein constituted the default conduit strategy whenever anatomically appropriate. Within the cryoanalgesia cohort, 27 patients underwent bilateral internal mammary artery grafting, whereas 3 patients received a single left internal mammary artery graft. The number of distal coronary anastomoses differed between study groups, with more distal grafts in the cryoanalgesia cohort (3.3 ± 0.7 vs. 2.8 ± 0.7; *p* = 0.007).

**Figure 2:**
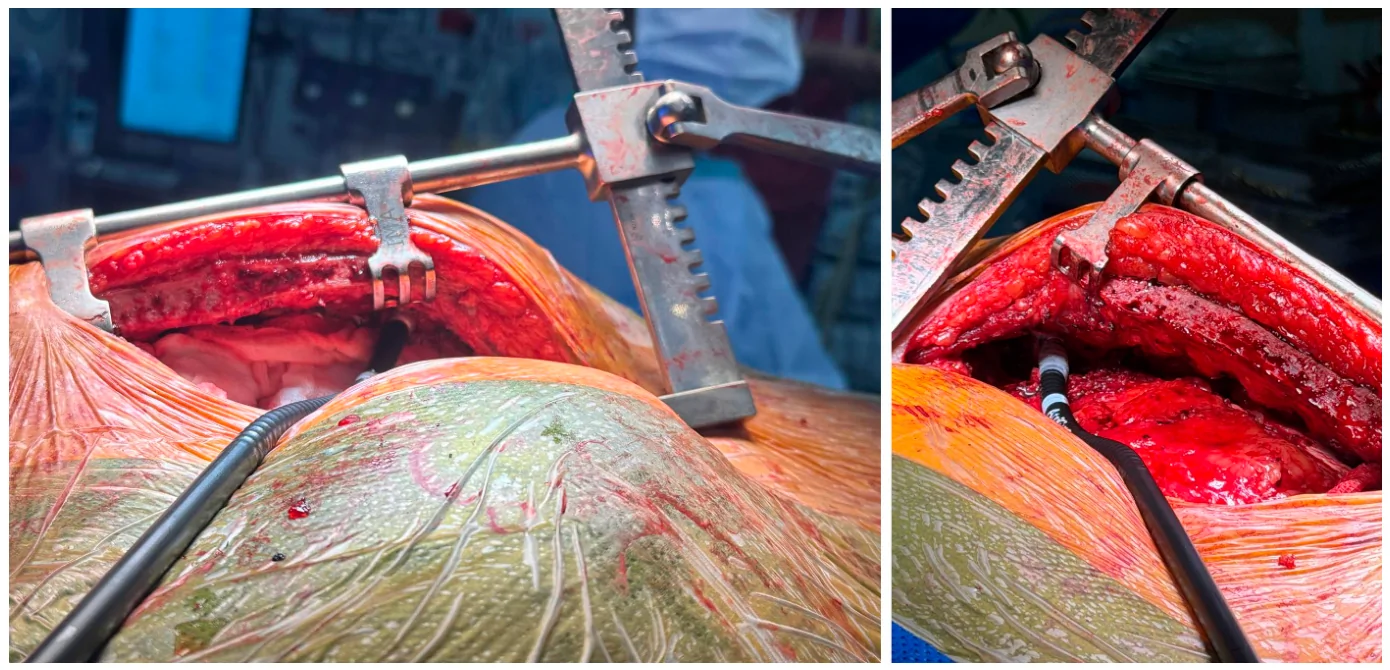
Representative intraoperative photographs demonstrating bilateral intercostal cryoanalgesia performed under direct vision during isolated coronary artery bypass grafting through median sternotomy. (**Left**) CryoSPHERE^®^ MAX probe (AtriCure, Mason, OH, USA) applied to the left second–seventh intercostal nerves following internal mammary artery harvest. (**Right**) Corresponding application of the CryoSPHERE^®^ MAX probe (AtriCure, Mason, OH, USA) to the right intercostal nerves. Cryoablation was performed approximately 2–4 cm lateral to the internal mammary artery using the manufacturer’s recommended application protocol.

Following completion of internal mammary artery harvest, bilateral intercostal cryoanalgesia was performed before commencement of CABG using the CryoSPHERE^®^ MAX probe (AtriCure, Mason, OH, USA). In patients undergoing bilateral internal mammary artery grafting, cryoanalgesia was performed immediately following bilateral internal mammary artery harvest. In patients receiving a single left internal mammary artery graft, the ipsilateral pleura was deliberately opened to facilitate exposure of the intercostal nerves. The probe was applied directly under vision to the second through seventh intercostal nerves bilaterally, approximately 2–4 cm lateral to the internal mammary artery, with each nerve treated sequentially according to the manufacturer’s recommended application protocol (Figure 2).

The CryoSPHERE^®^ MAX probe, with a 10-mm ball tip, was placed against each target intercostal neurovascular bundle for a single 60-s freeze cycle, with a target displayed probe-temperature range of −50 °C to −70 °C. Six nerves (second through seventh) were treated sequentially on each side. The console temperature was monitored throughout application; after active defrost, the probe was not moved until its displayed temperature was above 0 °C and tissue adhesion had released. Adequate treatment was judged by continuous direct tissue contact and formation of a visible ice ball throughout the cycle. Twelve applications required 12 min of active freezing and approximately 20 min in total, including positioning, handling and nerve identification. The procedure was performed by one of two experienced operators using the same technique. No intraoperative technical event or immediate postoperative event attributed by the treating team to cryoanalgesia was documented. The evaluation did not prospectively grade sensory change, dysaesthesia, neuropathic pain or chronic post-sternotomy pain.

### 2.4. Perioperative Analgesia

Both groups received the institution’s routine multimodal pathway: intraoperative opioid analgesia at the anaesthetist’s discretion, postoperative intravenous morphine patient-controlled analgesia (PCA), regular paracetamol unless contraindicated, and additional oral or rescue analgesia according to clinical need. PCA was discontinued and oral treatment introduced when pain was controlled, oral intake was tolerated and the clinical team considered intravenous rescue no longer necessary. Because this was a service evaluation of routinely delivered care, the source dataset did not preserve induction-maintenance drug doses, uniform PCA bolus/lockout settings, all non-opioid doses, or a protocolised discontinuation time for every patient. These unmeasured differences remain a potential source of confounding.

Cryoanalgesia was intended as an adjunct to, rather than a replacement for, the background pathway. However, adherence to every component of perioperative analgesia was not prospectively audited; equivalence of all co-interventions therefore cannot be assumed.

### 2.5. Outcome Measures

The prespecified primary endpoint was cumulative movement-evoked postoperative pain burden, quantified as AUC from numerical rating-scale scores recorded on postoperative days 1, 2 and 3. Rest-pain AUC was a secondary endpoint. No formal sample-size calculation was performed because the cohort size was set pragmatically for this initial service evaluation.

Pain was recorded once on each of postoperative days 1, 2 and 3, at rest and during routine movement, using an 11-point numerical rating scale from 0 (no pain) to 10 (worst imaginable pain). The specific movement manoeuvre and clock time were not standardised, assessors were members of the clinical team, and neither patients nor assessors were blinded. Assessor training beyond routine institutional practice was not documented. Patient-level AUC was calculated by the trapezoidal rule across the three daily observations. These features permit measurement and reporting bias.

Secondary endpoints were rest-pain AUC, pain trajectories, PCA opioid consumption, total observed opioid consumption, time to first bowel opening, postoperative length of stay and the correlation between pain burden and opioid exposure. Administered opioids were converted to milligrams of OME using the institution’s standard equianalgesic conversion approach. Total observed consumption extended to discharge rather than a common fixed postoperative time; because intervention patients had shorter stays, this endpoint is susceptible to unequal exposure time. PCA consumption is therefore also reported separately, and all opioid findings are interpreted as exploratory associations.

### 2.6. Early Health-Economic Evaluation

An exploratory break-even calculation was undertaken from the hospital-provider perspective. The documented acquisition price of the disposable CryoSPHERE^®^ MAX probe was £1500 per patient. This was compared with the observed adjusted association with postoperative length of stay; it was not treated as a causal or cash-releasing saving.

The bed-day value required to offset the probe price was calculated as £1500 divided by the adjusted length-of-stay difference. Sensitivity analysis used both the point estimate (1.32 days) and its 95% confidence limits (0.71–1.93 days), giving break-even bed-day values of approximately £1136, £2113 and £777, respectively. The calculation did not include the approximately 20 min of additional operating-room, anaesthesia, perfusion or staff time; equipment, maintenance or training; opioid/PCA savings; complications; readmissions; or downstream costs. It therefore estimates only a preliminary accounting threshold, not cost-effectiveness, budget impact or realised savings.

### 2.7. Statistical Analysis

Continuous variables are presented as mean ± standard deviation or median (interquartile range) according to data distribution, whereas categorical variables are summarised as frequencies and percentages.

Between-group comparisons were performed using the independent-samples Student’s *t* test or Mann–Whitney *U* test for continuous variables and the χ^2^ test or Fisher’s exact test for categorical variables, as appropriate.

Repeated pain measurements were analysed using linear mixed-effects models with treatment group, postoperative day (categorical) and treatment-by-day interaction as fixed effects and a patient-specific random intercept. Model coefficients were estimated with 95% confidence intervals. Given the small sample, these models were regarded as exploratory; residual plots were reviewed for gross departures from linear-model assumptions.

AUC outcomes and length of stay were analysed using identity-link Gaussian regression. Because opioid consumption was right-skewed, inference for its adjusted mean difference used non-parametric bootstrap confidence intervals (2000 patient-level resamples); time to first bowel opening was analysed with an identity-link Gaussian model to retain interpretation in days. To limit overfitting, each adjusted model included treatment group and number of distal grafts, the operative variable with the largest baseline imbalance. No data-driven variable selection was used. Adjustment cannot remove residual confounding, particularly unmeasured temporal, provider and co-intervention effects.

Effect sizes were quantified using Cohen’s d. Pearson correlation described the relationship between movement-pain AUC and total observed opioid consumption. Complete-case analysis was identical to the primary analysis because outcome data were complete. Bootstrap inference was used for the skewed opioid endpoint. Owing to the sample size and unavailable group-specific dates and surgeon data, additional calendar-time and provider-adjusted models were not estimable.

All adjusted associations are reported as adjusted mean differences with corresponding 95% confidence intervals (CIs), with emphasis placed on estimation of the magnitude and uncertainty of the observed between-group differences rather than statistical significance alone. Statistical significance was defined as a two-sided *p* value < 0.05.

All statistical analyses were performed using R version 4.5.0 (R Foundation for Statistical Computing, Vienna, Austria).

## 3. Results

### 3.1. Study Population

Between 1 November 2025 and 30 May 2026, the analytic sample comprised 60 patients undergoing isolated CABG through median sternotomy: 30 received bilateral intercostal cryoanalgesia and 30 received standard multimodal analgesia. The cohort included 53 men and 7 women. Complete pain and early recovery outcomes were available for these patients. Group-specific calendar dates and surgeon distribution were unavailable.

### 3.2. Baseline Characteristics

Baseline characteristics are summarised in Table 1. The cohorts were not well matched: the cryoanalgesia group received more distal grafts (SMD 0.73; *p* = 0.007), and potentially important imbalances were present for hypertension (SMD 0.41), good left ventricular function (SMD 0.40), three-vessel disease (SMD 0.36) and previous myocardial infarction (SMD 0.34). These differences reinforce the risk of residual confounding.

All patients underwent isolated off-pump coronary artery bypass grafting through median sternotomy. In every case, the pleura were opened and drains were placed within the opened pleural cavities. In the cryoanalgesia group, 27 patients received bilateral internal mammary artery grafts and three received a single left internal mammary artery graft; supplementary conduits comprised endoscopically harvested long saphenous vein. The cryoanalgesia group received more distal coronary anastomoses than the standard-analgesia group (3.3 ± 0.7 versus 2.8 ± 0.7; *p* = 0.007). All procedures were performed by the two study surgeons (S.G.R. and A.K.). Operative duration and postoperative outcomes, including ventilation duration, intensive care stay, complications and readmissions, were not collected as part of this service evaluation and therefore could not be compared between groups.

### 3.3. Postoperative Pain Outcomes

**Figure 3:**
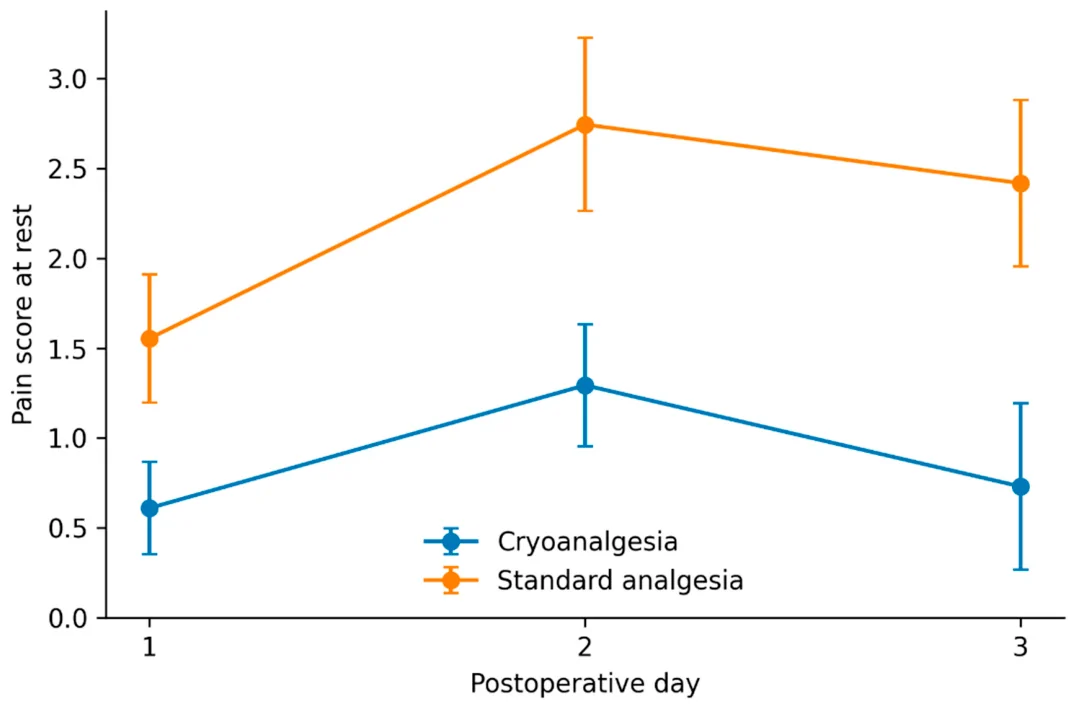
Mean postoperative pain scores at rest on postoperative days 1–3 after isolated CABG. Points show group means and error bars show standard errors.

**Figure 4:**
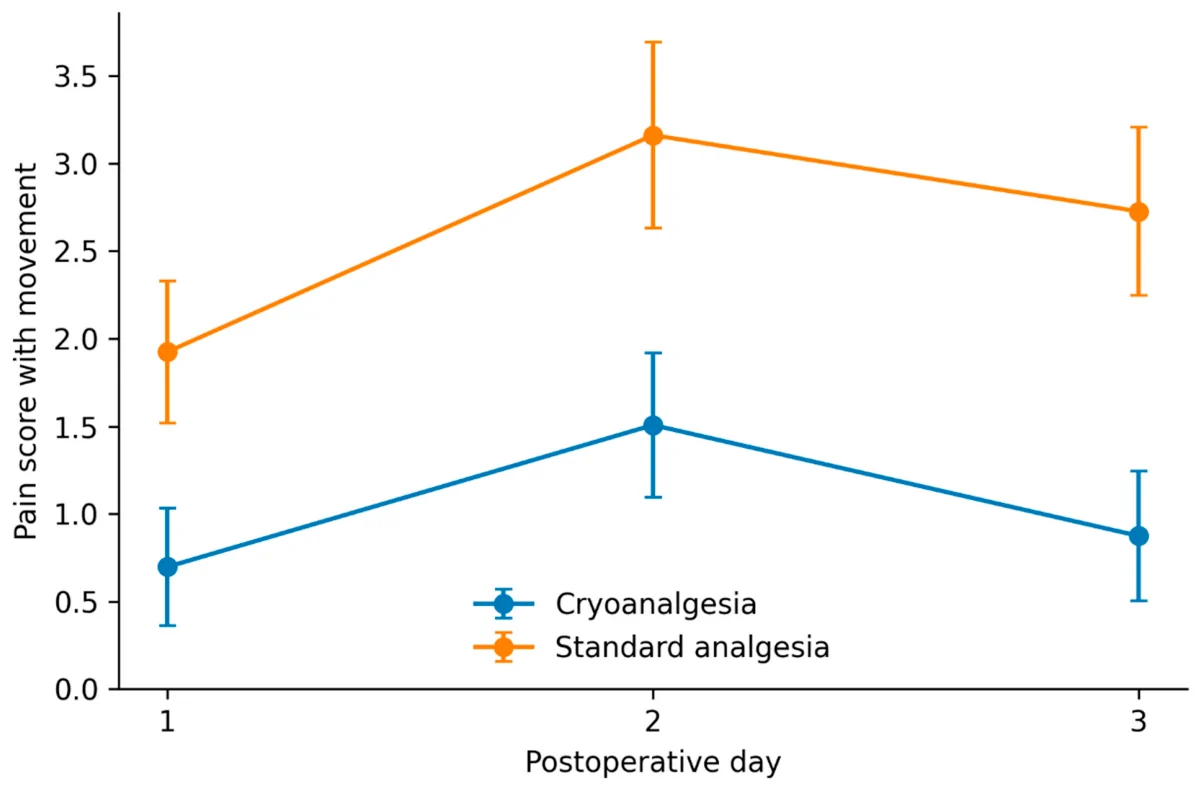
Mean movement-evoked postoperative pain scores on postoperative days 1–3 after isolated CABG. Points show group means and error bars show standard errors.

Bilateral intercostal cryoanalgesia was associated with consistently lower postoperative pain scores at every assessment point both at rest and during movement (Table 2; Figure 3 and Figure 4). The magnitude of benefit increased throughout the early postoperative period, with large effect sizes observed across all pain assessments.

On postoperative day 1, pain at rest was reduced by 0.94 points (95% confidence interval [CI], −1.39 to −0.49; *p* < 0.001), while movement-evoked pain was reduced by 1.23 points (95% CI, −1.76 to −0.69; *p* < 0.001). Similar improvements were maintained on postoperative days 2 and 3. Cumulative postoperative pain burden, quantified by area under the curve (AUC), was substantially lower in the cryoanalgesia group. Rest pain AUC was reduced by 2.77 score-days (95% CI, −3.73 to −1.80; *p* < 0.001), whereas movement pain AUC was reduced by 3.20 score-days (95% CI, −4.22 to −2.18; *p* < 0.001), corresponding to very large effect sizes (Cohen’s *d* = 1.47 and 1.64, respectively).

Linear mixed-effects modelling was directionally consistent with the AUC analyses. Pain at rest showed group, postoperative-day and group-by-day associations, whereas movement-evoked pain showed group and postoperative-day associations without clear evidence of a group-by-day interaction. These exploratory repeated-measures findings do not establish an independent causal effect because allocation was non-random and relevant co-interventions were incompletely measured.

### 3.4. Opioid Consumption and Early Recovery

**Figure 5:**
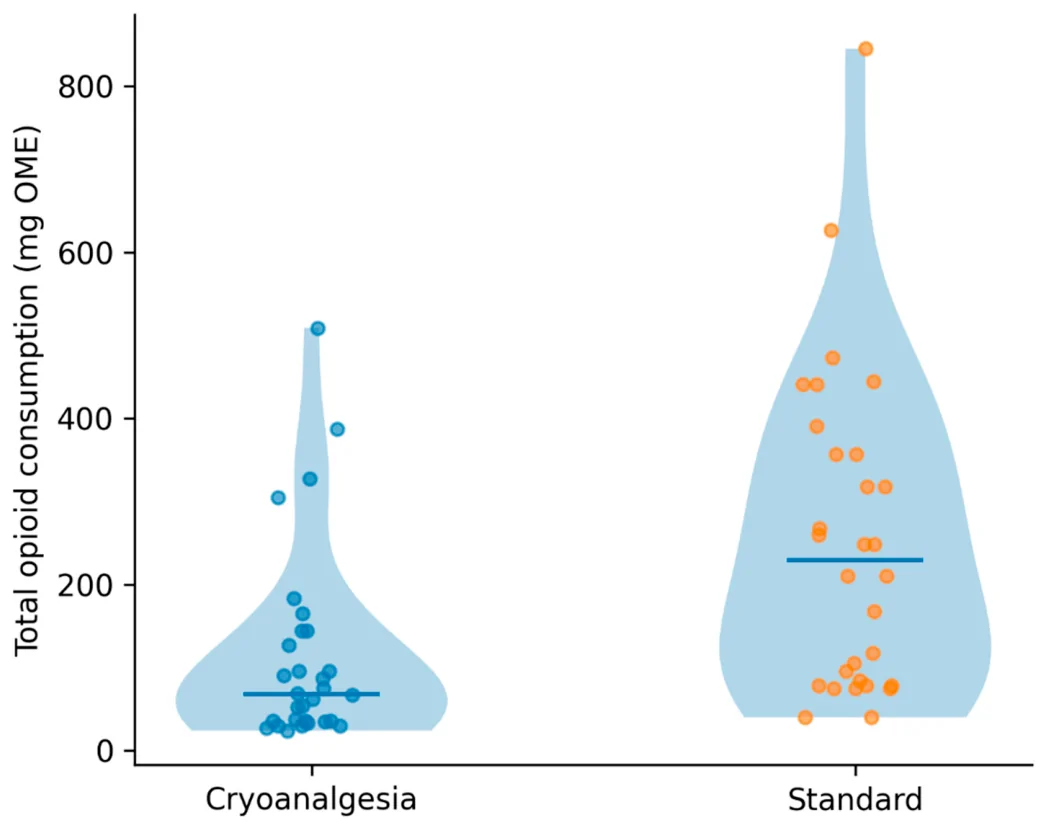
Violin plots demonstrating the distribution of total postoperative opioid consumption expressed as oral morphine equivalents (OME) in patients receiving bilateral intercostal cryoanalgesia and standard multimodal analgesia.

**Figure 6:**
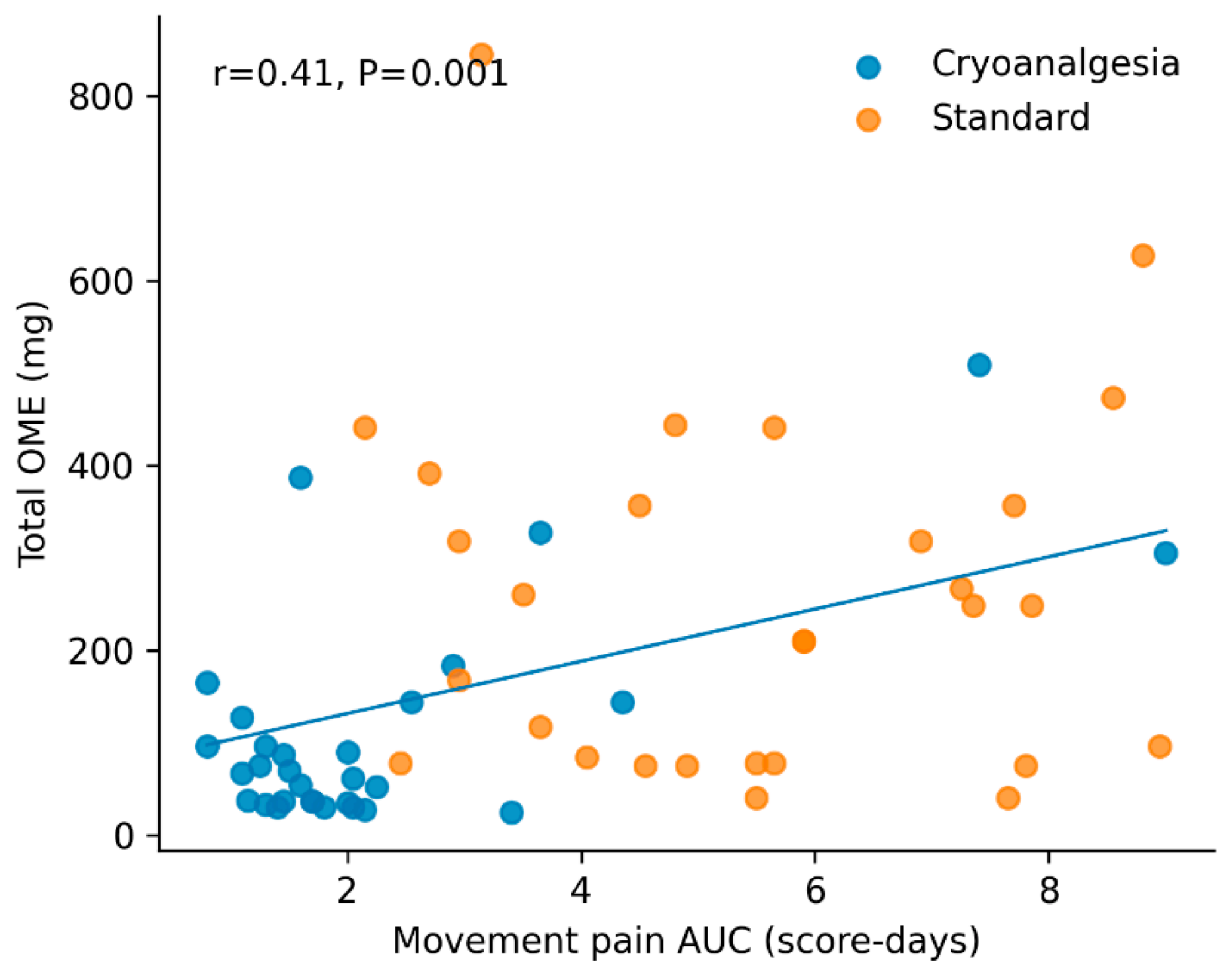
Correlation between movement-pain AUC and total observed opioid consumption. Each point represents one patient; the line is the fitted linear regression (Pearson r = 0.41; *p* = 0.001).

Lower postoperative pain scores were observed alongside lower opioid requirements and measures of earlier recovery (Table 3; Figure 5 and Figure 6). PCA opioid consumption was lower by 110.4 mg oral morphine equivalents (OME) (95% CI −163.72 to −57.11; *p* < 0.001), while total observed postoperative opioid consumption was lower by 139.1 mg OME (95% CI −222.27 to −55.83; *p* = 0.002). These between-group differences should be interpreted as exploratory associations. Across the entire study population, cumulative movement pain burden, quantified by AUC, demonstrated a moderate positive correlation with total postoperative opioid consumption (Pearson r = 0.41, *p* = 0.001), indicating that patients experiencing greater cumulative postoperative pain required higher opioid doses (Figure 6).

Patients undergoing bilateral intercostal cryoanalgesia achieved earlier return of bowel function (2.6 ± 0.7 vs. 3.2 ± 0.8 days; mean difference −0.67 days; 95% CI −1.07 to −0.27; *p* = 0.001) and experienced a significantly shorter postoperative hospital stay (5.0 ± 0.8 vs. 6.6 ± 1.4 days; mean difference −1.57 days; 95% CI −2.16 to −0.97; *p* < 0.001).

### 3.5. Adjusted Associations

**Figure 7:**
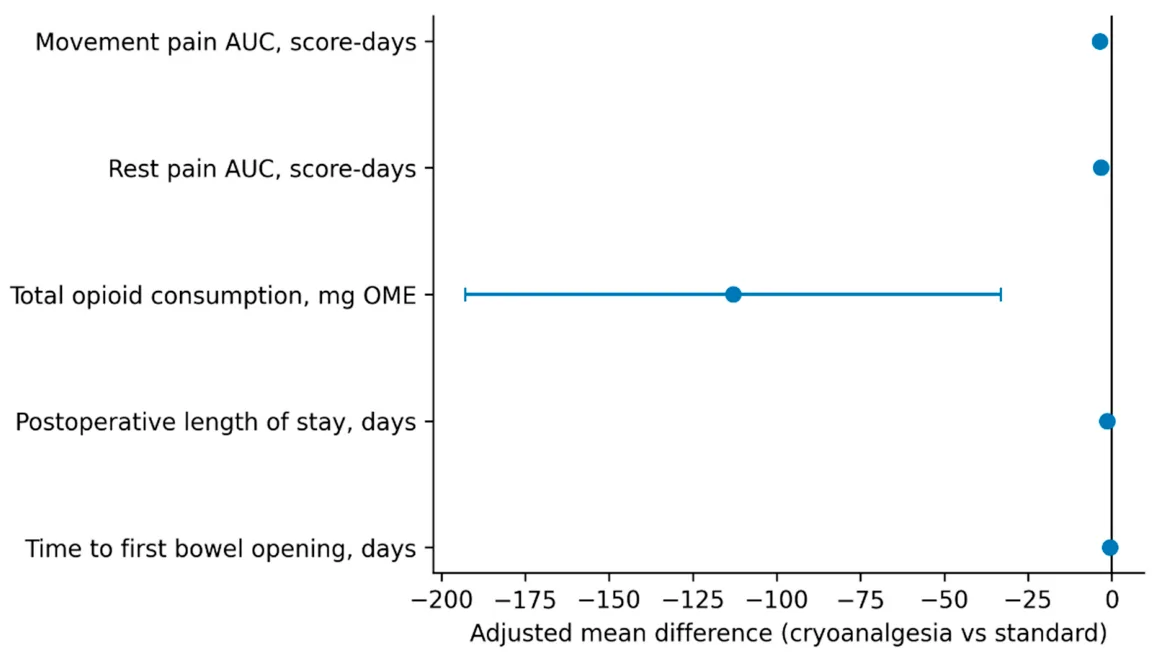
Forest plot of exploratory adjusted mean differences associated with cryoanalgesia. Horizontal bars denote 95% confidence intervals.

In parsimonious exploratory models adjusted for number of distal grafts, cryoanalgesia was associated with lower pain AUC, lower total observed opioid consumption, shorter postoperative stay and earlier bowel opening (Table 4; Figure 7). These estimates represent adjusted associations and should not be interpreted as independent or causal effects.

The largest adjusted associations were observed for movement-pain AUC and opioid consumption (Figure 7). Small-sample uncertainty, non-random allocation and unmeasured temporal, provider and co-intervention effects remain important despite covariate adjustment.

### 3.6. Early Health-Economic Evaluation

**Figure 8:**
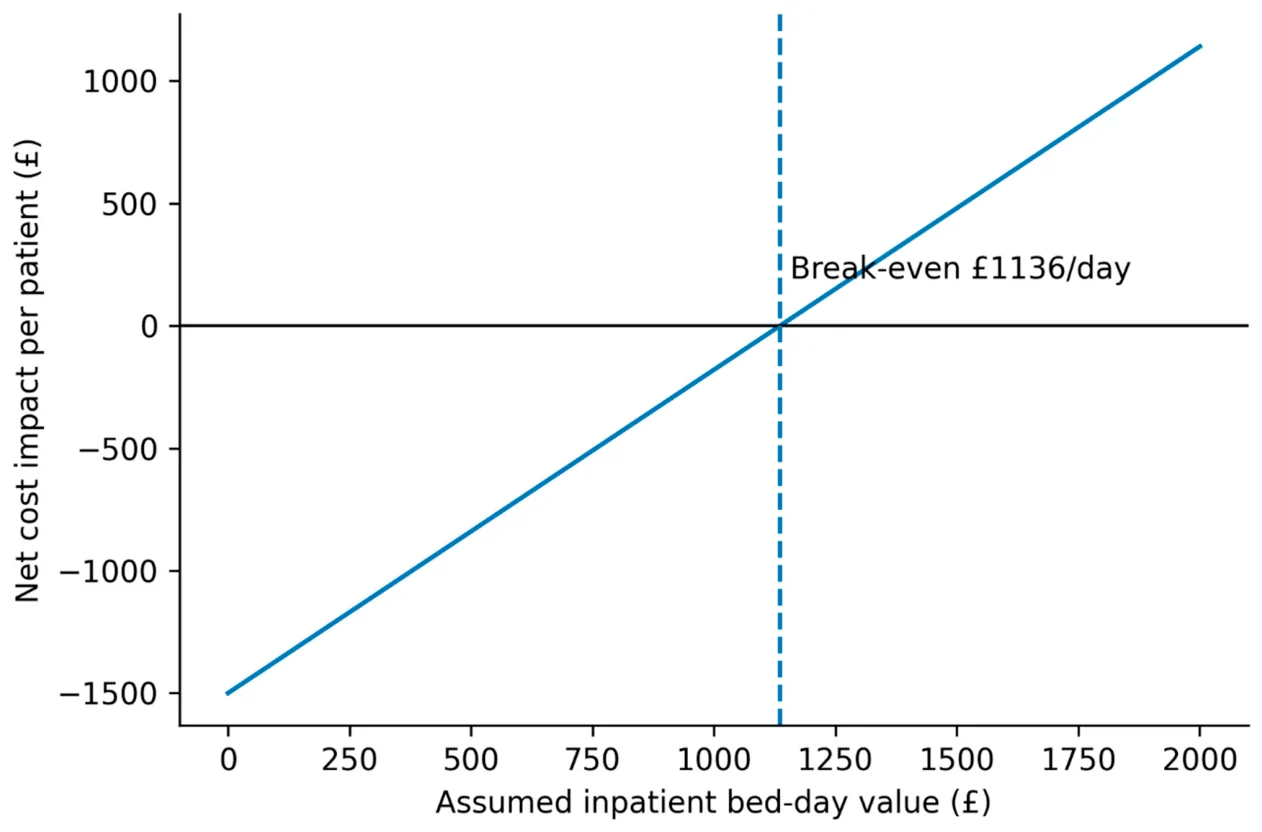
Preliminary break-even relationship between assumed inpatient bed-day value and net accounting offset using the adjusted length-of-stay point estimate. This is not a cost-effectiveness or budget-impact analysis.

The preliminary break-even calculation used the £1500 probe price and the adjusted 1.32-day length-of-stay difference (Table 5; Figure 8). The point-estimate threshold was approximately £1136 per bed-day. Using the 95% confidence limits for the length-of-stay association (0.71–1.93 days), the corresponding threshold ranged from approximately £2113 to £777 per bed-day. These figures exclude additional procedural and downstream costs and do not establish actual savings or cost-effectiveness.

## 4. Discussion

This small, non-randomised comparative service evaluation found that bilateral intercostal cryoanalgesia was associated with lower early pain scores, reduced observed opioid consumption, earlier bowel opening and shorter postoperative stay. The consistency of unadjusted and exploratory adjusted estimates provides a preliminary signal, but it does not distinguish an observed association from selection, temporal, provider or perioperative-management differences. The break-even calculation is hypothesis-generating and should not be interpreted as evidence of cost saving or cost-effectiveness.

The principal finding of this study is that bilateral intercostal cryoanalgesia was associated with lower cumulative postoperative pain following median sternotomy. Rather than relying on isolated pain scores, we selected area under the curve (AUC) as the primary endpoint because it provides an integrated measure of the patient’s overall pain experience during the postoperative recovery period [22]. This approach captures both the magnitude and duration of pain and is less susceptible to temporal variation than single time-point assessments. The concordance between the AUC analyses and linear mixed-effects modelling indicates that the observed between-group difference in pain was present throughout the early postoperative period rather than being confined to individual assessments [23].

Several mechanistic considerations may explain the magnitude and consistency of the analgesic benefit observed. Sternotomy pain is generated by multiple sources, including sternal osteotomy, periosteal injury, bilateral pleural opening, internal mammary artery harvest and chest drain placement, all of which converge through the anterior intercostal nerves [4,5,24]. Bilateral cryoanalgesia directly interrupts transmission through these nerves at the time of surgery, before central sensitisation becomes established, thereby attenuating the amplification of postoperative nociceptive signalling [25]. Unlike single-injection regional techniques, which are limited by the duration of local anaesthetic action, cryoanalgesia provides prolonged sensory blockade throughout the period of greatest postoperative pain while avoiding catheter displacement, local anaesthetic toxicity and the need for repeated intervention [26]. The sustained divergence of pain trajectories demonstrated by both the area-under-the-curve analysis and repeated-measures modelling is consistent with this prolonged physiological effect and provides a plausible explanation for the accompanying reductions in opioid requirements, earlier gastrointestinal recovery and shorter hospitalisation observed in the present study.

Several characteristics distinguish bilateral intercostal cryoanalgesia from alternative regional analgesic techniques. Cryoanalgesia is performed under direct vision by the operating surgeon, eliminating dependence on ultrasound guidance and avoiding the technical variability associated with fascial plane blocks. Because analgesia results from reversible axonotmesis with preservation of the connective tissue architecture, prolonged sensory blockade is achieved without indwelling catheters or continuous local anaesthetic infusion [27]. This may be particularly advantageous in cardiac surgical patients, in whom perioperative anticoagulation and antiplatelet therapy can complicate catheter-based regional techniques. Furthermore, cryoanalgesia does not rely on postoperative catheter maintenance, reducing nursing workload and the potential for catheter-related failure.

The reduction in opioid consumption observed in this study is clinically important. Opioid-related adverse effects, including nausea, vomiting, ileus, constipation, urinary retention, sedation and respiratory depression, remain major impediments to enhanced recovery after cardiac surgery [7]. Reduced opioid exposure may facilitate earlier mobilisation, improve participation in physiotherapy and contribute to the earlier restoration of gastrointestinal function observed in the present study. Although the study was not designed to evaluate opioid-related complications, the significant reduction in oral morphine equivalent requirements suggests that bilateral intercostal cryoanalgesia has the potential to become an important component of opioid-sparing perioperative care pathways. In an era of increasing awareness of persistent postoperative opioid use, strategies that safely reduce perioperative opioid exposure are of growing importance.

The observed length-of-stay difference warrants cautious interpretation. Discharge is influenced by complications, ventilation and intensive-care duration, rehabilitation, readmission risk, discharge criteria, bed flow and organisational practice, none of which was comprehensively modelled here. Moreover, an avoided occupied bed-day is not necessarily a cash-releasing saving. The preliminary calculation omitted additional operating-room, anaesthesia, perfusion and staff time; equipment, maintenance and training; opioid and PCA costs; complications; readmissions; and downstream resource use. A prospective trial with patient-level resource collection and uncertainty analysis is required before economic claims can be made.

Beyond its analgesic effects, bilateral intercostal cryoanalgesia may represent an enabling intervention within contemporary enhanced recovery pathways for cardiac surgery. Current ERAS recommendations emphasise multimodal, opioid-sparing analgesia to facilitate early extubation, mobilisation and discharge, yet few regional techniques have been adopted widely because of technical complexity, anticoagulation concerns or limited duration of action [9,20,26]. Cryoanalgesia addresses several of these limitations by providing surgeon-delivered, long-lasting regional analgesia without postoperative catheter management. Although confirmation in larger prospective studies is required, the present findings suggest that cryoanalgesia could become an important component of future ERAS protocols for median sternotomy.

This study has important limitations. It was a single-centre, non-randomised service evaluation of only 60 patients, and allocation was not governed by a documented uniform rule. Exclusion of patients who declined intervention-specific consent, pragmatic control selection, unavailable group-specific dates and incomplete provider information create substantial risks of selection, consent, temporal and implementation bias. Baseline imbalance was clinically relevant, including an SMD of 0.73 for distal graft number; small-sample adjustment cannot eliminate residual or unmeasured confounding and may yield unstable estimates. The cohort included 53 men and 7 women, limiting sex-specific inference and generalisability. Pain scores were subjective; assessments were unblinded, the movement manoeuvre and clock time were not standardised, and reporting bias is possible. Perioperative co-interventions and operative characteristics were incompletely captured. Total observed opioid exposure was measured to discharge rather than over a fixed period and may be biased by differential length of stay. Complications, intensive-care duration, readmissions and discharge determinants were not sufficiently complete for adjusted analysis. Safety ascertainment was limited to documented immediate events; persistent numbness, sensory change, dysaesthesia, neuropathic pain and chronic post-sternotomy pain were not assessed. Finally, the preliminary break-even calculation was not a cost-effectiveness analysis and excluded major procedural and downstream cost categories. The findings should therefore be considered hypothesis-generating.

## 5. Conclusions

In this preliminary non-randomised service evaluation, bilateral intercostal cryoanalgesia during CABG was associated with lower early postoperative pain and opioid exposure and faster recovery. Causal benefit, safety beyond discharge and cost-effectiveness cannot be established from these data. A sufficiently powered multicentre randomised trial with standardised analgesia and pain assessment, fixed opioid-observation windows, comprehensive operative and complication reporting, longer-term sensory and chronic-pain follow-up, and prospective health-economic evaluation is warranted.

## Figures and Tables

**Table 1:** Baseline demographic and clinical characteristics.

Characteristic	Cryoanalgesia (n = 30)	Standard Analgesia (n = 30)	*p* Value	SMD
Age, years	63.6 ± 10.8	63.1 ± 7.0	0.835	0.05
Male sex	27 (90.0)	26 (86.7)	1.000	0.10
Body mass index, kg/m^2^	29.5 ± 5.5	29.0 ± 4.8	0.696	0.10
Diabetes mellitus	11 (36.7)	11 (36.7)	1.000	0.00
Hypertension	15 (50.0)	21 (70.0)	0.187	0.41
Chronic pulmonary disease	1 (3.3)	1 (3.3)	1.000	0.00
Previous myocardial infarction	15 (50.0)	20 (66.7)	0.295	0.34
Previous PCI	10 (33.3)	8 (26.7)	0.779	0.15
Good left ventricular function (ejection fraction ≥ 50%)	21 (70.0)	26 (86.7)	0.209	0.40
Three-vessel disease	23 (76.7)	18 (60.0)	0.267	0.36
Elective surgery	15 (50.0)	15 (50.0)	1.000	0.00
Number of distal grafts	3.3 ± 0.7	2.8 ± 0.7	0.007	0.73

**Table 2:** Postoperative pain outcomes.

Outcome	Cryoanalgesia (n = 30)	Standard Analgesia (n = 30)	Mean Difference (95% CI)	*p* Value	Cohen’s *d*
Day 1 pain at rest	0.6 ± 0.7	1.6 ± 1.0	−0.94 (−1.39 to −0.49)	<0.001	1.08
Day 1 pain with movement	0.7 ± 0.9	1.9 ± 1.1	−1.23 (−1.76 to −0.69)	<0.001	1.20
Day 2 pain at rest	1.3 ± 0.9	2.7 ± 1.3	−1.45 (−2.05 to −0.85)	<0.001	1.24
Day 2 pain with movement	1.5 ± 1.2	3.2 ± 1.5	−1.66 (−2.34 to −0.97)	<0.001	1.25
Day 3 pain at rest	0.7 ± 1.3	2.4 ± 1.3	−1.69 (−2.35 to −1.02)	<0.001	1.31
Day 3 pain with movement	0.9 ± 1.0	2.7 ± 1.3	−1.85 (−2.47 to −1.23)	<0.001	1.55
Rest pain AUC (score-days)	2.0 ± 1.7	4.7 ± 2.0	−2.77 (−3.73 to −1.80)	<0.001	1.47
Movement pain AUC (score-days)	2.3 ± 1.8	5.5 ± 2.1	−3.20 (−4.22 to −2.18)	<0.001	1.64

**Table 3:** Opioid consumption and recovery outcomes.

Outcome	Cryoanalgesia (n = 30)	Standard Analgesia (n = 30)	Mean Difference (95% CI)	*p* Value	Cohen’s *d*
PCA opioid consumption (mg OME)	74.9 ± 56.1	185.3 ± 133.0	−110.42 (−163.72 to −57.11)	<0.001	1.08
Total observed opioid consumption (mg OME)	113.1 ± 119.4	252.1 ± 192.8	−139.05 (−222.27 to −55.83)	0.002	0.87
Time to first bowel opening (days)	2.6 ± 0.7	3.2 ± 0.8	−0.67 (−1.07 to −0.27)	0.001	0.86
Postoperative length of stay (days)	5.0 ± 0.8	6.6 ± 1.4	−1.57 (−2.16 to −0.97)	<0.001	1.37

**Table 4:** Exploratory adjusted associations with cryoanalgesia.

Outcome	Adjusted Mean Difference	95% CI	*p* Value
Movement pain AUC (score-days)	−3.56	−4.62 to −2.50	<0.001
Rest pain AUC (score-days)	−3.16	−4.19 to −2.14	<0.001
Total opioid consumption (mg OME)	−112.95	−192.77 to −33.14	0.006
Postoperative length of stay (days)	−1.32	−1.93 to −0.71	<0.001
Time to first bowel opening (days)	−0.49	−0.92 to −0.06	0.027

**Table 5:** Preliminary break-even accounting calculation using the adjusted length-of-stay point estimate.

Assumed Inpatient Bed-Day Value (£)	Adjusted LOS Reduction (Days)	Gross Bed-Day Cost Offset (£)	Probe Cost (£)	Net Cost Impact (£)
500	1.32	660	1500	−840
750	1.32	990	1500	−510
1000	1.32	1320	1500	−180
1200	1.32	1584	1500	+84
1500	1.32	1980	1500	+480

## Data Availability

The data presented in this study are available on request from the corresponding author. The data are not publicly available due to privacy or ethical restrictions.

## Abbreviations

The following abbreviations are used in this manuscript:

AUC	Area under the curve
BMI	Body mass index
CABG	Coronary artery bypass grafting
CI	Confidence interval
ERAS	Enhanced Recovery After Surgery
GDPR	General Data Protection Regulation
LOS	Length of stay
OME	Oral morphine equivalents
PCA	Patient-controlled analgesia
PCI	Percutaneous coronary intervention
REC	Research Ethics Committee
SMD	Standardised mean difference
STROBE	Strengthening the Reporting of Observational Studies in Epidemiology
UK	United Kingdom
